# Author Correction: Structure of a type IV secretion system core complex encoded by multi-drug resistance F plasmids

**DOI:** 10.1038/s41467-022-30584-1

**Published:** 2022-05-16

**Authors:** Xiangan Liu, Pratick Khara, Matthew L. Baker, Peter J. Christie, Bo Hu

**Affiliations:** 1 Department of Microbiology and Molecular Genetics, McGovern Medical School, 6431 Fannin St, Houston, TX 77030 USA; 2 Department of Biochemistry and Molecular Biology, McGovern Medical School, 6431 Fannin St, Houston, TX 77030 USA

**Keywords:** Bacterial secretion, Bacterial structural biology, Cryoelectron microscopy

Correction to: *Nature Communications* 10.1038/s41467-022-28058-5, published online 19 January 2022.

The original version of this Article contained an error in the Acknowledgements section, which incorrectly omitted the following:

‘We gratefully thank the UTHealth Houston CryoEM Core for use of their facilities and microscopes’.

This has been corrected in both the PDF and HTML versions of the Article.

