# Supplementary material for: Structure of a type IV secretion system core complex encoded by multi-drug resistance F plasmids
Source: Nat Commun. 2022 Jan 19;13:379. doi: 10.1038/s41467-022-28058-5 (PMC8770708; doi:10.1038/s41467-022-28058-5)
Supplement: Supplementary file 1 — Supplementary Information [file 41467_2022_28058_MOESM1_ESM.pdf]

SUPPLEMENTARY INFORMATION

Structure of a Type IV Secretion System Core Complex Encoded by Multi-Drug Resistance F Plasmids

Xiangan Liu, Pratick Khara, Matthew L. Baker, Peter J. Christie, and Bo Hu

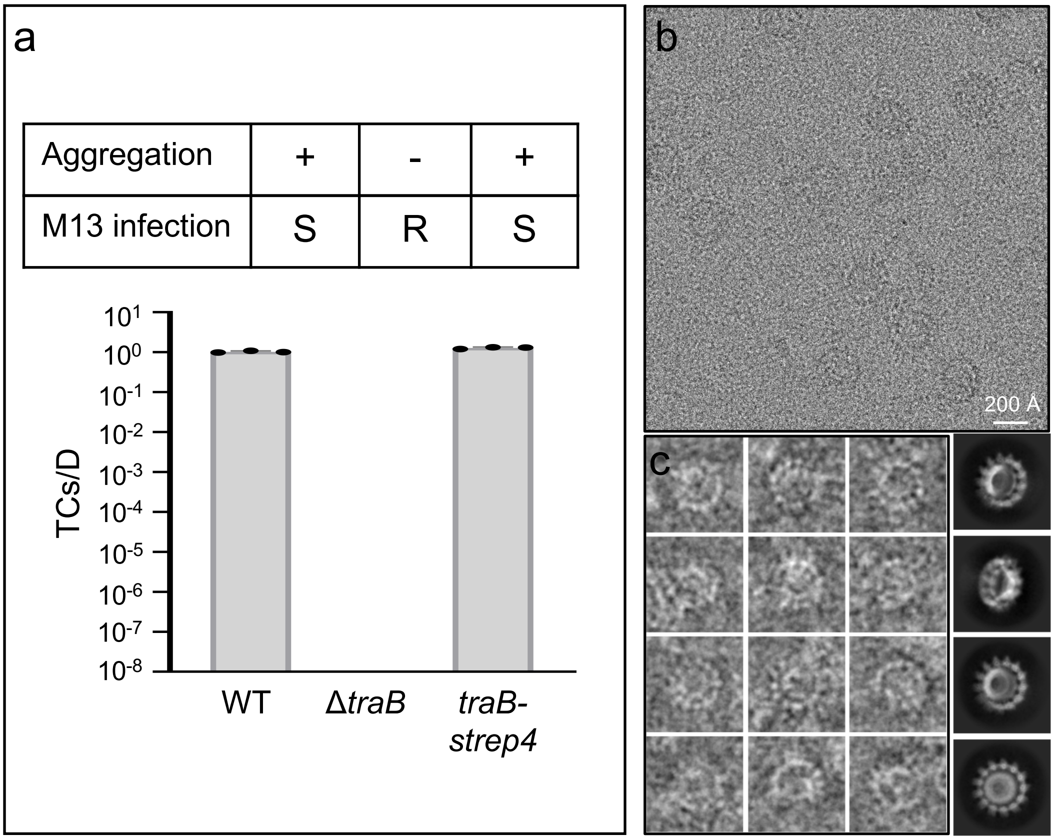

d

Description	Score	Coverage	# Peptides	# PSMs	# AAs	MW [kDa]
TraK	908.27	82.93	26	579	246	26.5
TraB	690.74	70.86	33	384	453	47.7
TraV	336.93	54.41	8	152	204	20.9
outer membrane porin protein C	162.8	59.13	22	98	367	40.3
outer membrane protein 3a	95.03	61.27	17	52	346	37.2
TraD	70.56	43.34	34	52	736	82.7
TraF	57.87	74.13	20	36	259	29.4
TrbI	56.22	66.23	11	26	154	16.8
TraG	36.02	33.89	25	31	965	104.0
TraH	35.10	49.01	22	36	453	48.9
TraT	11.09	24.49	5	9	245	26.0
TraN	8.51	14.91	6	6	617	66.2
TraE	2.05	32.09	5	5	187	21.3

Supplementary Figure 1. Cryo-EM data collection and 2D analysis. a) *E. coli* MC4100*(*pED208-*traB*$_{Strep4}$) phenocopies the isogenic strain harboring the wild-type (WT) pED208 plasmid, as determined by F pilus-mediated aggregation (+/-) and M13 phage sensitivity (R/S), and F plasmid transfer (TCs/D; Transconjugants per Donor). Experiments were replicated three times in triplicate. Plasmid transfer frequencies are reported as transconjugants per donor (TCs/D). Experiments were performed in triplicate and individual replicates are indicated as dots, the mean of the three replicates are represented by bar heights, and standard deviations by error bars. **b)** A representative image of F-encoded T4SS particles visualized by Cryo-EM. Scale bar 200 Å. **c)** Representative particles (left series) and 2D class averages (far right). See 'Methods' section for detailed information. **d)** LC-MS/MS mass spectrometry analysis of purified OMCCs from MC4100(pED208). TraK, TraB, and TraV predominated among the top 13 identified proteins. F-encoded Tra proteins detected in minor amounts were not visualized in the CryoEM map. Source data for the mating experiments are provided as a Source Data file.

Flowchart 1

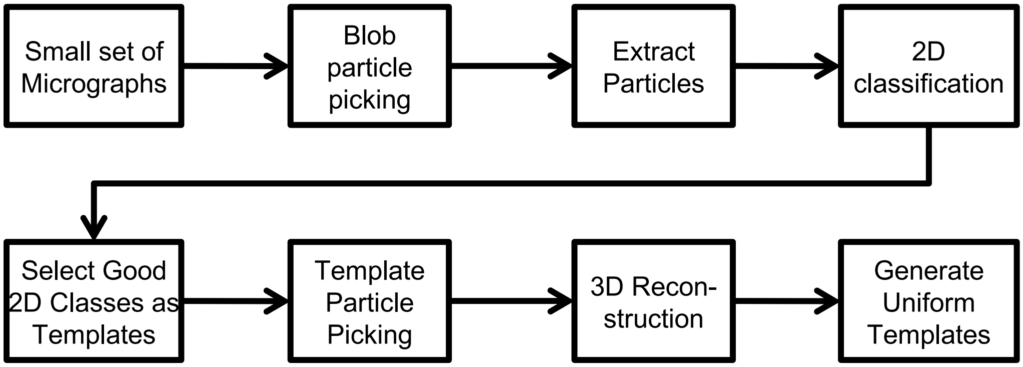

Flowchart 2

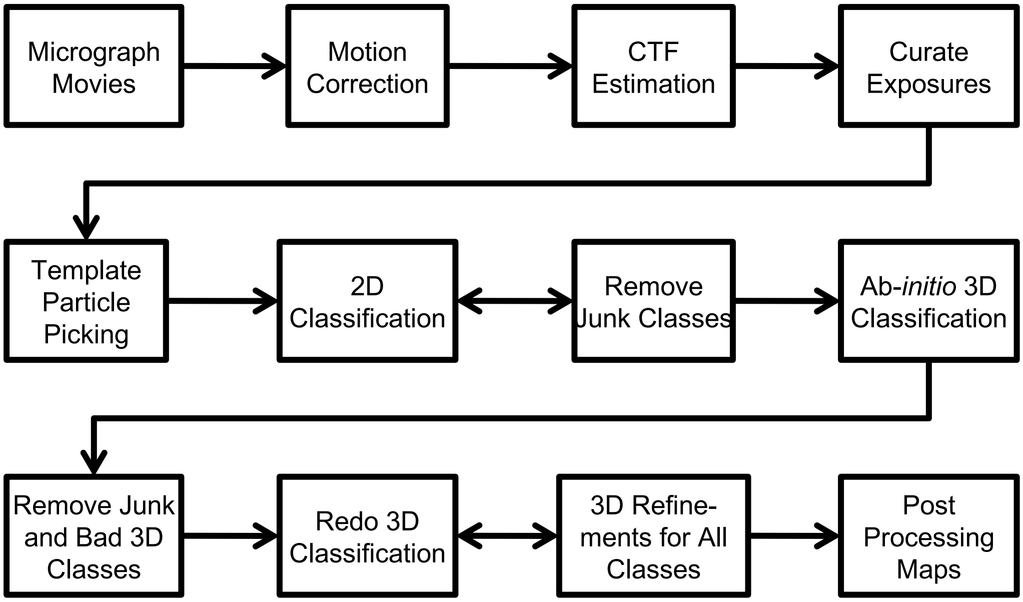

Supplementary Figure 2. Cryosparc v3.1.0 was used to process the data. Initial templates were generated according to Flowchart 1. Data were processing according to Flowchart 2.

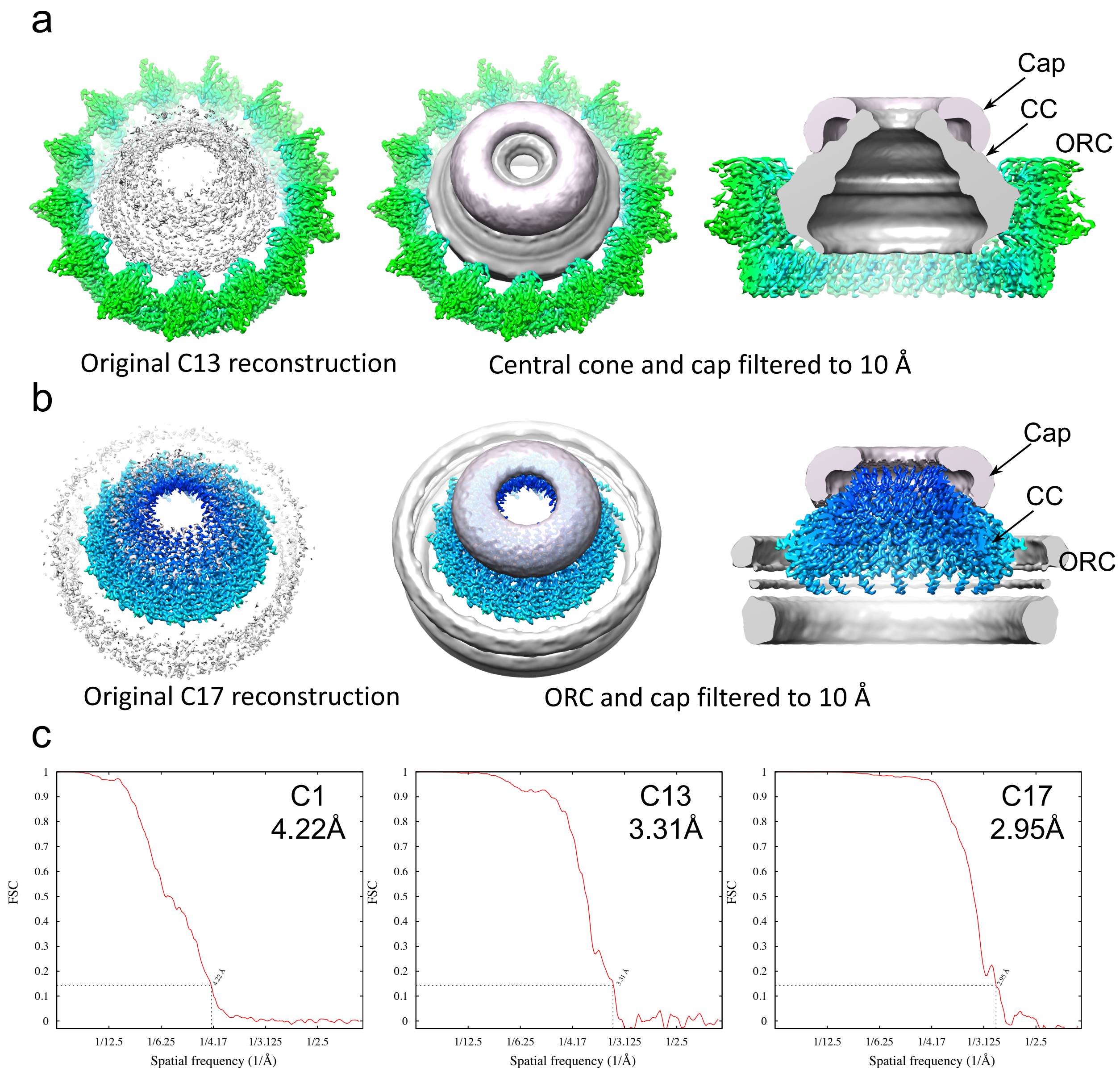

Supplementary Figure 3. **Symmetric reconstructions of the OMCC$_F$ from the pED208-carrying strain. a)** The outer ring complex (ORC) was reconstructed to 3.31 Å by applying C13 symmetry. **b)** The central cone (CC) was reconstructed to 2.95 Å by applying C17 symmetry. Cap densities were not resolved in either of the C13 or C17 maps. Colors: ORC (Green/aqua), CC (Blue shades), Unresolved densities (Gray). **c)** Fourier shell correlation (FSC) of C1, C13 and C17 reconstructions obtained from Cryosparc v3.1.0. Resolutions of the final maps were determined using FSCs at the 0.143 cutoff.

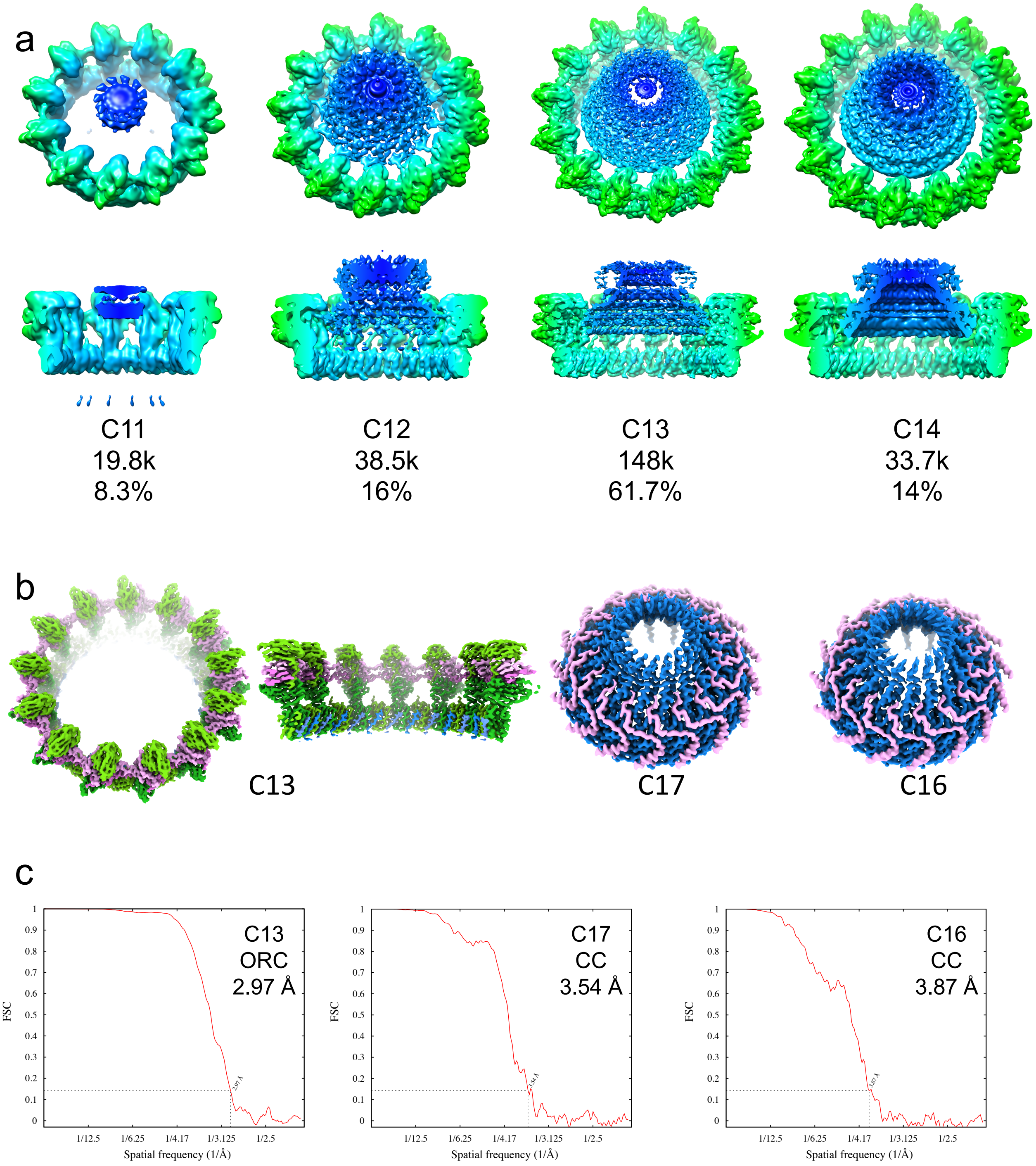

Supplementary Figure 4. Structure of OMCC from the TraV/K/B-producing strain. **a)** Top and central cut views of OMCCs exhibiting symmetry variations of 11- to 14-fold for the ORC substructure. The fold symmetry, numbers of particles, and proportions are shown for each class. Colors: Green/aqua shades, ORC; CC, blue shades. **b)** Left: Top and central cut view of the 13-fold ORC. Right: CC structures generated from the 13-fold symmetrical class of ORC particles exhibit 17- and 16-fold symmetries. Colors: TraK (Green), TraV (Pink), TraB (Blue). **c)** FSC of C13, C17 and C16 reconstructions. Resolutions of the final maps were determined using FSCs at the 0.143 cutoff.

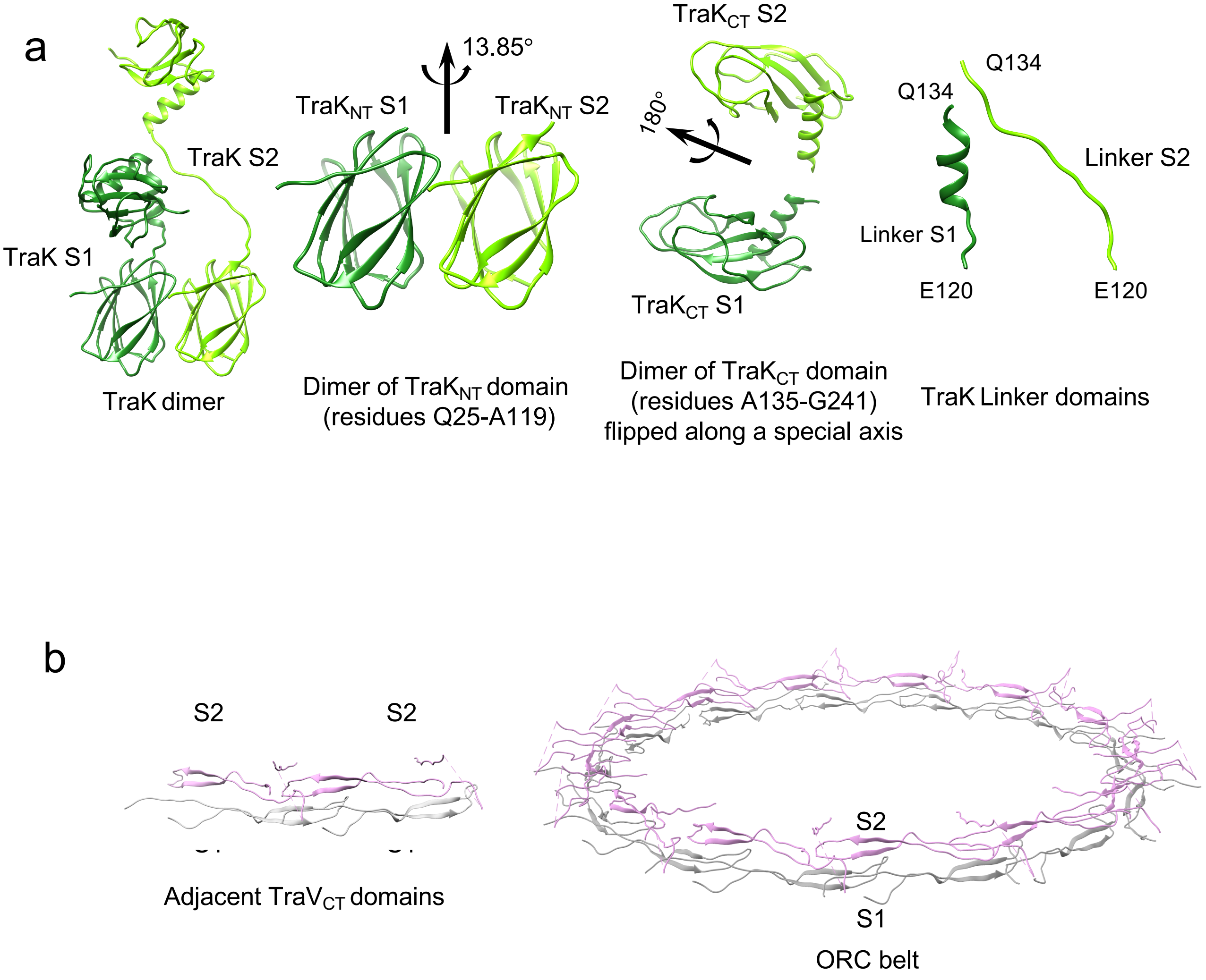

Supplementary Figure 5. Elements of the ORC structural unit. a) TraK dimer composed of subunits S1 and S2, and arrangements of the N- and C-terminal domain dimers. The linker domain of TraK subunit S1 is folded and detected in the high-resolution map, the linker of subunit S2 is extended and visible as a blurred density only when the map is filtered to 10 Å resolution. Dolors: TraK subunit 1 (dark green), TraK subunit 2 (light green). **b)** The C-terminal domain of TraV ($TraV_{CT}$, residues V150 - N204) is arranged as two antiparallel β-strands. C-terminal domains of the TraV S1 and S2 subunits stack on top of each to form a 4-stranded β-sheet that embeds into the $TraK_{CT}$ dimer in the ORC structural unit. $TraV_{CT}$ domains from the S2 subunits connect laterally to form the upper band while those from the S1 subunits form the lower band of the ORC belt that surrounds and stabilizes the $TraK_{CT}$ lobed-ring (see Fig. 2). Colors: TraV Subunit 1 (Gray), TraV subunit 2 (Pink).

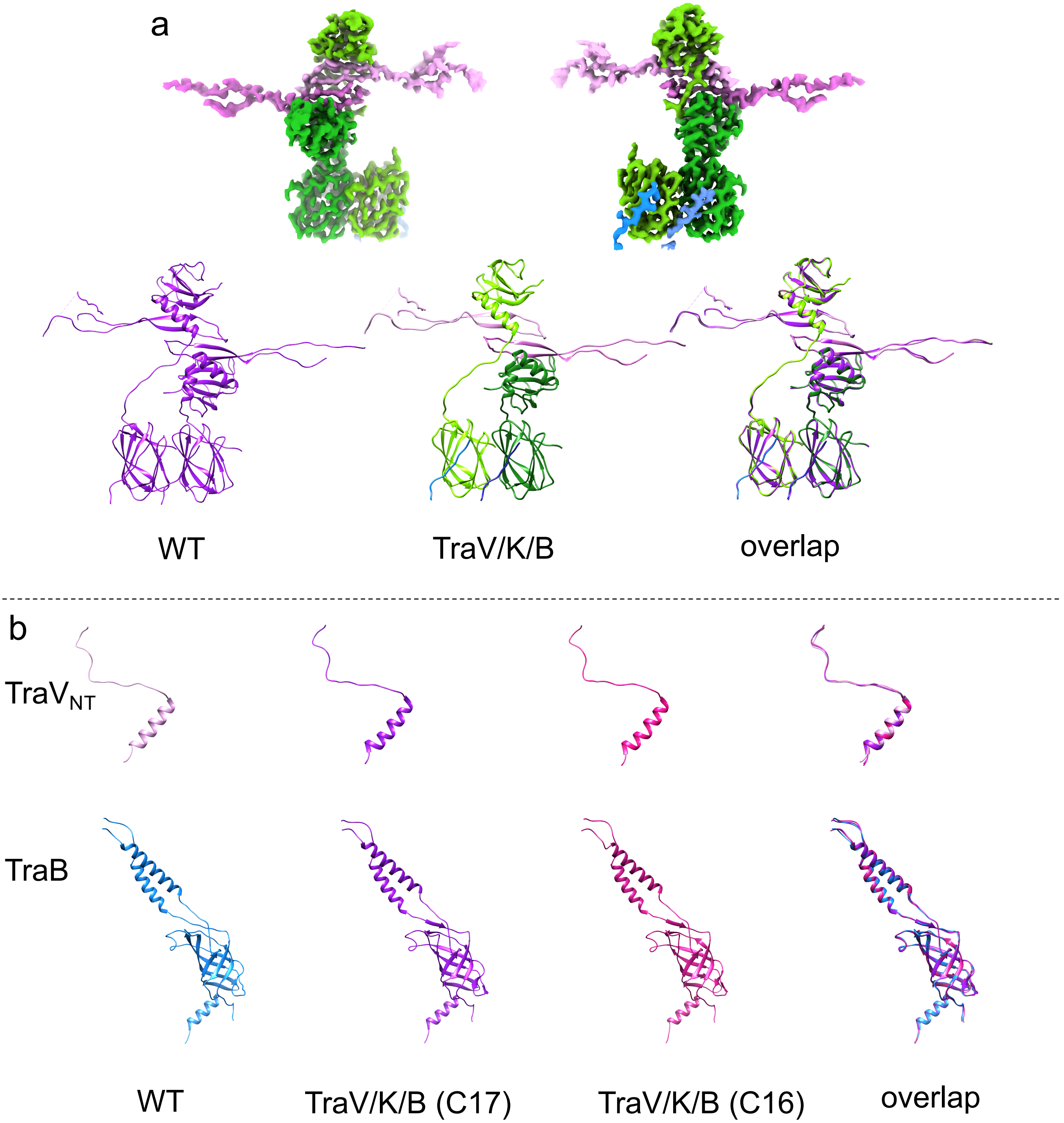

Supplementary Figure 6. Structural comparisons of OMCC components from the pED208-carrying (WT) and TraV/K/B-producing strains. a) Structural unit of the ORC. Upper: Outer and inner views of the structural unit from the TraV/K/B-producing strain. TraK monomers (dark and light green), TraV$_{CT}$ domains (dark and light purple), and TraB$_{R176\text{-}S186}$ linker segments (blue). Lower: Atomic models of the ORC structural unit from the WT and TraV/K/B-producing strains, with overlapping alignment of the two structural units. **b)** Structures of the TraV$_{NT}$ and TraB$_{\beta\text{-barrel/AP}}$ elements of the C17 CC from the WT strain and the C17 and C16 CCs from the TraV/K/B-producing strain, with overlapping alignments of the structures.

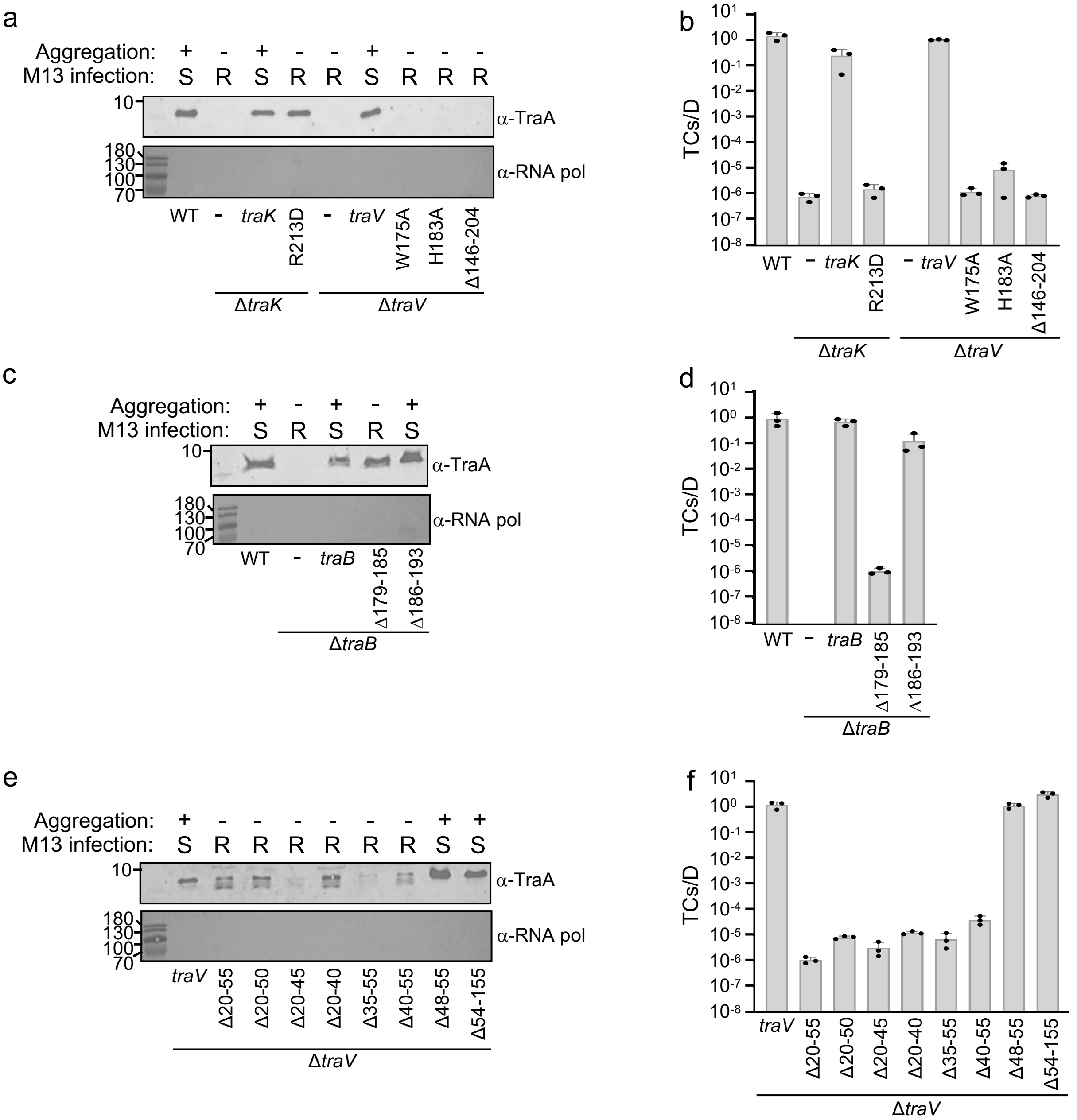

Supplementary Figure 7. Phenotypic characterization of *traK*, *traV*, and *traB* mutations. **a, b)** Strains: MC4100(pED208) (WT); MC4100 with pED208 deleted of *traK* (Δ*traK*) or *traV* (Δ*traV*) lacking (-) or expressing the *traK* or *traV* alleles listed. **a)** Tests for F pilus production, including F pilus-mediated cellular aggregation (Aggregation, +/-), susceptibility to infection by the F pilus-binding M13 bacteriophage (M13 infection, Sensitive/Resistant S/R), immunoblot analyses of extracellular fractions for detection of F pilus subunit TraA. Blots were developed with α-TraA antibody specific for pED208-encoded TraA pilin, and with α-RNA antibody specific for the β-subunit of *E.coli* RNA polymerase as a control for cell lysis. Molecular sizes (in kDa) of protein markers are listed at the left. **b)** Donor proficiency of MC4100 strains in 1.5 h liquid matings. Plasmid transfer frequencies are reported as transconjugants per donor (TCs/D). Experiments were performed in triplicate and individual replicates are indicated as dots, the mean of the three replicates are represented by bar heights, and standard deviations by error bars. **Panels c - f:** Analyses were repeated as described above for strains harboring Δ*traB* or Δ*traV* mutations lacking (-) or expressing the alleles listed. Source data are provided as a Source Data file.

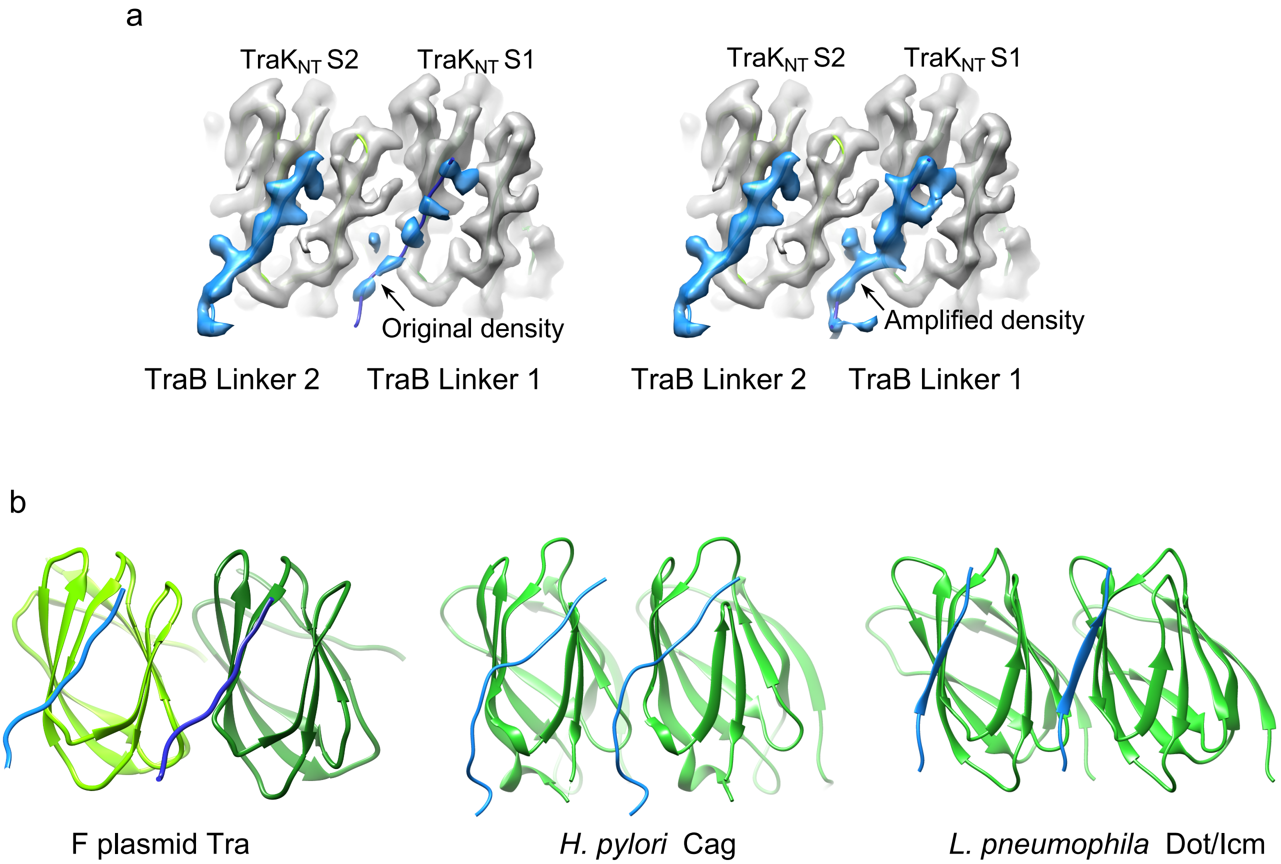

Supplementary Figure 8. TraB Linker - $TraK_{NT}$ interactions. **a)** Left: Inner view of the $TraK_{NT}$ dimer (Gray) and the original densities of the associated TraB Linker segments (residues R176-S186; Blue) in the C13 map, showing that TraB Linker 1 associated with $TraK_{NT}$ S1 is weaker than TraB Linker 2 associated with $TraK_{NT}$ S2. Right: Amplification of the TraB Linker 1 density. **b) Left:** TraB Linker - $TraK_{NT}$ dimer architecture compared with equivalent complexes in the *H. pylori* Cag and *L. pneumophila* Dot/Icm systems. In these systems, the N-terminal domains of TraK-like CagX and DotH dimerize and form specific contacts with Linker segments of TraB-like CagY and DotG, respectively. The Cag and Dot/Icm structures were generated from PDB files 6x6j and 6x65, respectively. Colors: TraKS1 (light green), TraKS2 (dark green), TraB linkers (light/dark blue), dimers of TraK/VirB9 homologs (green), linker segments of TraB/VirB10 homologs (blue).

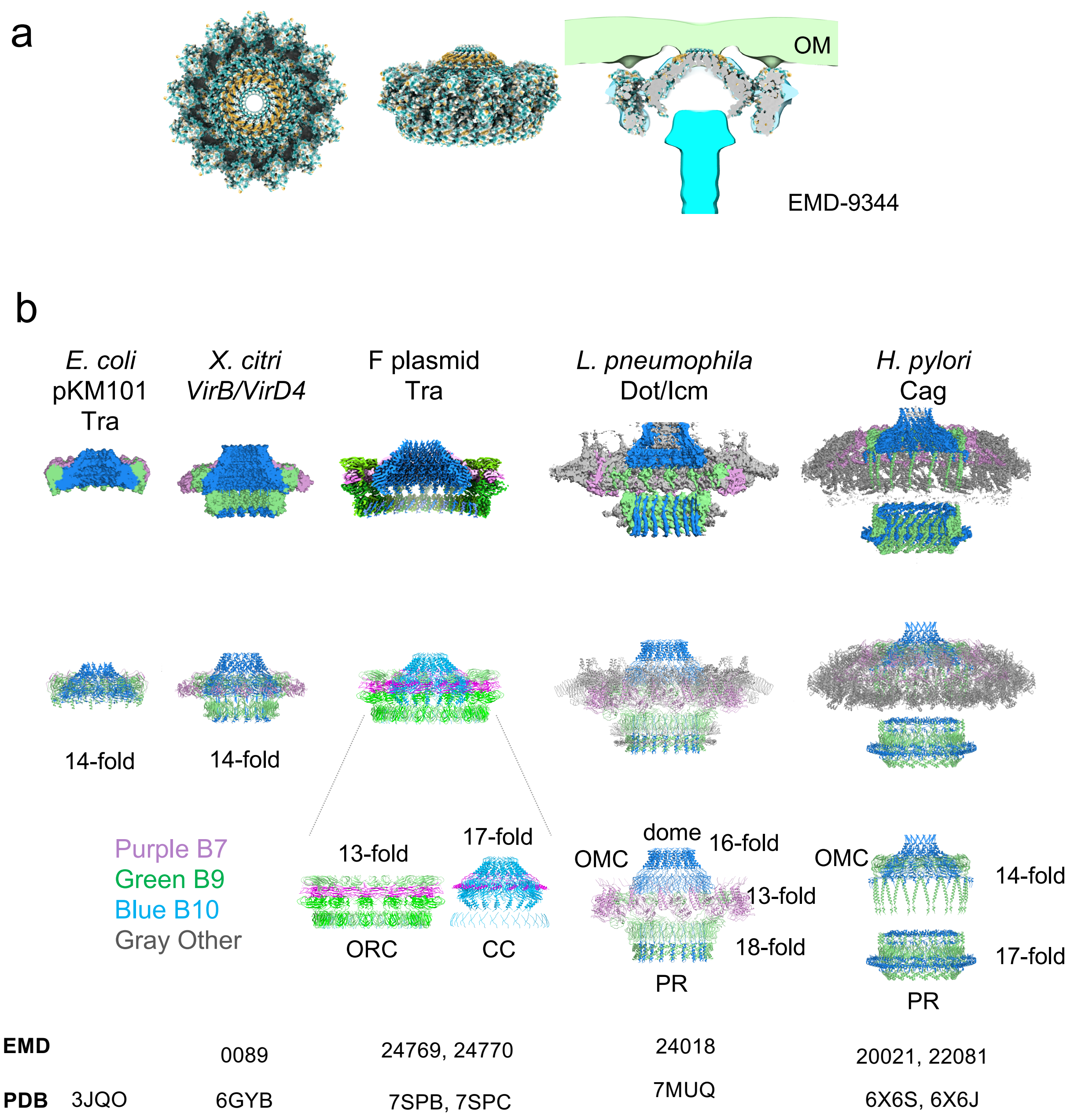

Supplementary Figure 9. Disposition of the OMCCF at the outer membrane and structural comparisons with OMCCs from the T4SS sources shown. a) Distribution of hydrophobic surface on the $OMCC_F$ structure and docking of the $OMCC_F$ into the in situ map of the $OMCC_F$ associated with outer membrane. Colors: Most hydrophilic (dark cyan), Most hydrophobic (dark goldenrod), Intermediate hydrophobicity (gray/white shades). (b) The *E. coli* pKM101 Tra and *X. citri* VirB/VirD4 T4SSs are examples of minimized systems, and the F plasmid Tra, *L. pneumophila* Dot/Icm and *H. pylori* Cag T4SSs are examples of expanded systems. Colors correspond to homologs or orthologs of the VirB7, VirB9, and VirB10 subunits from *A. tumefaciens*. Gray densities denote system-specific subunits. ORC, outer ring complex; CC, central cone; OMC, outer membrane cap; PR, periplasmic ring.

Supplementary Table 1. Strains, plasmids, and oligonucleotides used in these studies.

**Strains**	**Genotype**	**Source**
MC4100	F- *[araD139]B/r* Δ(*argF-lac*)*169* λ^- *e14*$^-$ *flhD5301* Δ(*fruK-yeiR*)*725*(*fruA25*) *relA1 rpsL150*(*strR*) *rbsR22* Δ(*fimB-fimE*)*632(::IS1) deoC1*	Christie lab
HME45	W3110 *gal490 pgl*Δ8 λ*c*I*857* Δ*(cro-bioA) rif*^{r}	(1)
DH5α	F– *endA1 glnV44 thi-1 recA1 relA1 gyrA96 deoR nupG purB20* φ80d*lacZ*ΔM15 Δ(*lacZYA-argF*)U169, hsdR17(*rK*$^-$*mK*$^+$), λ^-	Christie lab
BL21 (DE3)	F^-*ompT hsdS*$_B$(r_B^- m_B^-*) gal dcm* (DE3)	Christie lab

**Plasmids**	**Characteristics**	
pED208-Spec	Spec^R, *lac*$^+$, high-frequency transfer, hyperpiliated	(2)
pED208-Tet	Tet^R, *lac*$^+$, high-frequency transfer, hyperpiliated	(3)
pBAD24	Crb^r, pBAD cloning vector	(4)
pET-15(b)	Crb^r, *E. coli* T7 system expression vector, *lacI*^{q}	Novagen
pED208Δ*traK*	Tet^r, pED208 deleted of *traK*	This study
pED208-Δ*traB*:FRT-Kan^R-FRT	Tet^r Kan^r, pED208 with the FRT-Km^r-FRT cassette in place of *traB*	This study
pED208Δ*traB*	Tet^r, pED208 deleted of *traB*	This study
pED208-*traB*$_{Strep4}$	Tet^r, pED208 expressing *traB*$_{Strep4}$	This study
pED208Δ*traV*	Spc^r, pED208 deleted of *traV*	This study
pKBV	Crb^r, pET15(b) expressing *traK-traV* region of pED208*traB*$_{Strep4}$ from the T7 promoter	This study
pBAD24-*traK*	Crb^r, pBAD24 expressing *traK* from the P_{BAD} promoter	This study
pBAD24-*traK*_R213D	Crb^r, pBAD24 expressing P_{BAD}::*traK*_R213D	This study
pBAD24-*traB*	Crb^r, pBAD24 expressing P_{BAD}::*traB*	This study
pBAD24-up-*traB*-dn	Crb^r, pBAD24 containing *traB* and ~600-bp of 5’ and 3’ flanking regions	This study
pBAD24-up-*traB*$_{Strep4}$-dn	Crb^r, pBAD24 expressing P_{BAD}::*traB*$_{Strep4}$	This study
pBAD24-*traB*Δ179-185	Crb^r, pBAD24 expressing *traB*Δ179-185 from the P_{BAD} promoter	This study
pBAD24-*traB*Δ186-193	Crb^r, pBAD24 expressing P_{BAD}::*traB*Δ186-193	This study
pBAD24-*traV*	Crb^r, pBAD24 expressing P_{BAD}::*traV*	This study
pBAD24-*traV*Δ20-55	Crb^r, pBAD24 expressing P_{BAD}::*traV*Δ20-55	This study
pBAD24-*traV*Δ20-50	Crb^r, pBAD24 expressing P_{BAD}::*traV*Δ20-50	This study
pBAD24-*traV*Δ20-45	Crb^r, pBAD24 expressing P_{BAD}::*traV*Δ20-45	This study
pBAD24-*traV*Δ20-40	Crb^r, pBAD24 expressing P_{BAD}::*traV*Δ20-40	This study
pBAD24-*traV*Δ35-55	Crb^r, pBAD24 expressing P_{BAD}::*traV*Δ35-55	This study
pBAD24-*traV*Δ40-55	Crb^r, pBAD24 expressing P_{BAD}::*traV*Δ40-55	This study
pBAD24-*traV*Δ48-55	Crb^r, pBAD24 expressing P_{BAD}::*traV*Δ48-55	This study
pBAD24-*traV*Δ54-155	Crb^r, pBAD24 expressing P_{BAD}::*traV*Δ54-155	This study
pBAD24-*traV*Δ146-204	Crb^r, pBAD24 expressing P_{BAD}::*traV*Δ146-204	This study

pBAD24-*traV*_W175A	Crb^r, pBAD24 expressing P_{BAD}:: *traV*_W175A	This study
pBAD24-*traV*_H183A	Crb^r, pBAD24 expressing P_{BAD}:: *traV*_H183A	This study

**Oligonucleotides**	**Sequences (5′ to 3′)**	**Purpose**
AA9_traK_delF	CGGAGGCTTTACCCGTATCGGTCGGTTC TATGAGGTGACGAATGAAAAATAACAT TCCGGGGATCCGTCGACC	Deletion of *traK*
AA10_traK_delR	TCCCCGTTTCCACGGATAACGGTGACGG TCATTCTTCCTCCGCCCATCAGAGTTGT AGGCTGGAGCTGCTTCG	Deletion of *traK*
AA31_traKchk_F	CAATGTCTCGCCTGAAACGG	Confirmation of Δ*traK*
AA32_traKchk_R	CACGAATGCGATCCTGACTG	Confirmation of Δ*traK*
AA23_traKCl_F	AACTAGCTAGCAGGAGGAATTCACCAT GAAAAATAACCTTCCGGCGT	Cloning of *traK* in pBAD24
AA24_traKcl_R	AACCCAAGCTTTTATTAACGTTGGCCAT CTTCACC	Cloning of *traK* in pBAD24
traK_R213D_F	GATGCCGTGATGTTTGATAACAACGCA CAGAC	Construction of *traK* mutants in pBAD24
traK_R213_R	CACCCCGGGTTTCCAGAAGTCCTG	Construction of *traK* mutants in pBAD24
AA11_traB_delF	CGTCACCGTTATCCGTGGAAACGGGGA GGG TGAAGATGGCCAACGTTAATAAAATTC CGGGGATCCGTCGACC	Deletion of *traB*
AA12_traB_delR	TTGTGTTGTTGTGGTCGGTTTCCCGGCC CCGTTTACTGTGTGACGACCTGTCCTGT AGGCTGGAGCTGCTTCG	Deletion of *traB*
AA33_traBchk_F	GCCCTCACGGCCTGAAAC	Confirmation of Δ*traB*
AA34_traBchk_R	GTACCCGCTGACACCCAGTG	Confirmation of Δ*traB*
traB-NheI_F	CTAGCTAGCAGGAGGAATTCACCATGG CCAACGTTAATAAAGTCGTCC	Cloning of *traB* in pBAD24
traB-HindIII_R	CCCAAGCTTTTACTGTGTGACGACCTGT CCG	Cloning of *traB* in pBAD24
AA141_traB_L_NH_F	AAACTAGCTAGCGGCGGCCAGTTCCGG CTGAG	Construction of pBAD24-up-*traB*-dn
AA142_traB_L_Hd_R	AAACCCAAGCTTGTCGCTCTCTGTGGCA CCAACAC	Construction of pBAD24-up-*traB*-dn
Twin_Strep_F	AGCGCTTGGAGCCACCCGCAGTTCGAA AAAGGTGGAGGTTCTGGCGGTGGATCG GGAGGTTCAGCGTGGAGCCACCCGCAG TTCGAGAAA	Construction of pBAD24-up-*traB* $_{Strep4}$-dn
Twin_Strep_R	TTTCTCGAACTGCGGGTGGCTCCACGCT GAACCTCCCGATCCACCGCCAGAACCT CCACCTTTTTCGAACTGCGGGTGGCTCC AAGCGCT	Construction of pBAD24-up-*traB* $_{Strep4}$-dn

iptraBL_F	TAAACGGGGCCGGGAAACCGACCA	Construction of pBAD24-up-*traB* $_{Strep4}$-dn
iptraBL_R	CTGTGTGACGACCTGTCCGGTCG	Construction of pBAD24-up-*traB* $_{Strep4}$-dn
traB_rec_F	GCCGTGATGTTTGATAACAACGCACAGAC	Construction of pED208-*traB* $_{Strep4}$
traB_rec_R	CCGCCCATCCCTTTCTCGTCTTC	Construction of pED208-*traB* $_{Strep4}$
traB_186_F	AGTCTGCCGGATACCGGGCC	Construction of *traB* mutants in pBAD24
traB_del179-185_R	TCCCGGACGCTGAATGGGGGTC	Construction of *traB* mutants in pBAD24
traB_del186-193_F	AAGCCCCGTTTCCCGTGGATTTCTTC	Construction of *traB* mutants in pBAD24
traB_del186-193_R	GAATTCCTGACTGTCCATCATTCCCGG	Construction of *traB* mutants in pBAD24
orb236	CCCGTCAGCCCTTTCAGGGCATAAATTCTCAGGAACACAGCGATGATTCCGGGGATCCGTCGACC	Deletion of *traV*
orb237	CGTTCAGAAATTTTTTCACCCGAAACTCCTTAATTTACCCTGGCCGGAAGTGTAGGCTGGAGCTGCTTCG	Deletion of *traV*
orb238	CTGGAGCCGTTACTGCATTCAC	Confirmation of Δ*traV*
orb239	GTCCAGACTGGCCAGATTGC	Confirmation of Δ*traV*
orb242	AGAGAGGCTAGCAGGAGGAATTCACCATGAAAAAAATCACACTTTTGCTGGC	Cloning of *traV* in pBAD24
orb243	AGAGAGAAGCTTTTAATTTACCCTGGCCGGAAGA	Cloning of *traV* in pBAD24
traV_del20-55_F	CCGGCTGCGGGCGGACTG	Construction of *traV* mutants in pBAD24
traV_del20-35_R	GGCACAACCGGATAACAGCAGGG	Construction of *traV* mutants in pBAD24
traV_del20-50_F	AAACAGGCGGGAAAGCCGGCTGC	Construction of *traV* mutants in pBAD24
traV_del20-45_F	CGGGACAAGGCGGCAAAACAGG	Construction of *traV* mutants in pBAD24
traV_del20-40_F	GCTAATCAACTGGCCCGGGACAAGG	Construction of *traV* mutants in pBAD24
traV_del35-55_R	GGTATCAGATGTTGTGGCATCGCAGTC	Construction of *traV* mutants in pBAD24
traV_del40-55_R	CGTCATGGTCATACAGGTATCAGATGTTG	Construction of *traV* mutants in pBAD24
traV_K48_R	GTCCCGGGCCAGTTGATTAGCC	Construction of *traV* mutants in pBAD24
traV_del54-155_F	GGAACTGTGCACCCACAACGCAGC	Construction of *traV* mutants in pBAD24
traV_del54-145_R	CGCCTGTTTTGCCGCCTTGTCC	Construction of *traV* mutants in pBAD24

traV_mut_F	TAAAAGCTTGGCTGTTTTGGCGGATGAGAG	Construction of *traV* mutants in pBAD24
traV_145_R	CGGTGTCGGTGTGACCACAGATAC	Construction of *traV* mutants in pBAD24
traV_W175A_F	GCAGTGGACAGCGATAACGCCTTCCATC	Construction of *traV* mutants in pBAD24
traV_W175_R	GGGCGCAATCCAGACCGTGGC	Construction of *traV* mutants in pBAD24
traV_H183A_F	GCACAGCCCGGTCGGGTGTCATTTG	Construction of *traV* mutants in pBAD24
traV_H183_R	GAAGGCGTTATCGCTGTCCACCC	Construction of *traV* mutants in pBAD24

Supplementary References

1. Thomason LC, Sawitzke JA, Li X, Costantino N, Court DL. 2014. Recombineering: genetic engineering in bacteria using homologous recombination. Curr Protoc Mol Biol 106:1 16 1-39.
2. Hu B, Khara P, Christie PJ. 2019. Structural bases for F plasmid conjugation and F pilus biogenesis in *Escherichia coli*. Proc Natl Acad Sci U S A 116:14222-14227.
3. Al Mamun AAM, Kishida K, Christie PJ. 2021. Protein transfer through an F plasmid-encoded type IV secretion system suppresses the mating-induced SOS response. mBio 12:e0162921.
4. Guzman LM, Belin D, Carson MJ, Beckwith J. 1995. Tight regulation, modulation, and high-level expression by vectors containing the arabinose PBAD promoter. J Bacteriol 177:4121-30.
