# Supplementary material for: Structure of a type IV secretion system core complex encoded by multi-drug resistance F plasmids
Source: Nat Commun. 2022 Jan 19;13:379. doi: 10.1038/s41467-022-28058-5 (PMC8770708; doi:10.1038/s41467-022-28058-5)
Supplement: Supplementary file 3 — Reporting Summary [file 41467_2022_28058_MOESM3_ESM.pdf]

nature portfolio

Corresponding author(s): Peter J Christie, Bo Hu

Last updated by author(s): Dec 1, 2021

Reporting Summary

Nature Portfolio wishes to improve the reproducibility of the work that we publish. This form provides structure for consistency and transparency in reporting. For further information on Nature Portfolio policies, see our Editorial Policies and the Editorial Policy Checklist.

Statistics

For all statistical analyses, confirm that the following items are present in the figure legend, table legend, main text, or Methods section.

n/a	Confirmed	
☐	☒	The exact sample size (*n*) for each experimental group/condition, given as a discrete number and unit of measurement
☐	☒	A statement on whether measurements were taken from distinct samples or whether the same sample was measured repeatedly
☒	☐	The statistical test(s) used AND whether they are one- or two-sided<br>*Only common tests should be described solely by name; describe more complex techniques in the Methods section.*
☒	☐	A description of all covariates tested
☒	☐	A description of any assumptions or corrections, such as tests of normality and adjustment for multiple comparisons
☐	☒	A full description of the statistical parameters including central tendency (e.g. means) or other basic estimates (e.g. regression coefficient) AND variation (e.g. standard deviation) or associated estimates of uncertainty (e.g. confidence intervals)
☒	☐	For null hypothesis testing, the test statistic (e.g. *F*, *t*, *r*) with confidence intervals, effect sizes, degrees of freedom and *P* value noted<br>*Give P values as exact values whenever suitable.*
☒	☐	For Bayesian analysis, information on the choice of priors and Markov chain Monte Carlo settings
☒	☐	For hierarchical and complex designs, identification of the appropriate level for tests and full reporting of outcomes
☒	☐	Estimates of effect sizes (e.g. Cohen's *d*, Pearson's *r*), indicating how they were calculated

Our web collection on statistics for biologists contains articles on many of the points above.

Software and code

Policy information about availability of computer code

Data collection	EPU software (FEI)
Data analysis	cryoSparc v.3.1.0, Phenix v.18.2, Coot v0.9.4.1, ChimeraX v1.2

For manuscripts utilizing custom algorithms or software that are central to the research but not yet described in published literature, software must be made available to editors and reviewers. We strongly encourage code deposition in a community repository (e.g. GitHub). See the Nature Portfolio guidelines for submitting code & software for further information.

Data

Policy information about availability of data

All manuscripts must include a data availability statement. This statement should provide the following information, where applicable:

- Accession codes, unique identifiers, or web links for publicly available datasets
- A description of any restrictions on data availability
- For clinical datasets or third party data, please ensure that the statement adheres to our policy

Reconstructed maps and atomic models are deposited to EMDB and PDB. The density maps have been deposited at the Electron Microscopy Data Bank with accession codes EMD-24768, EMD-24769, EMD-24770, EMD-24771, EMD-24772, EMD-24773, The atomic coordinates have been deposited at the Protein Data Bank with accession codes 7SPB, 7SPC, 7SPI, 7SPJ and 7SPK. Maps and Models will be available upon publication.

Field-specific reporting

Please select the one below that is the best fit for your research. If you are not sure, read the appropriate sections before making your selection.

- [x] Life sciences
- [ ] Behavioural & social sciences
- [ ] Ecological, evolutionary & environmental sciences

For a reference copy of the document with all sections, see nature.com/documents/nr-reporting-summary-flat.pdf

Life sciences study design

All studies must disclose on these points even when the disclosure is negative.

Sample size	For the OMCCF from the pED208-carrying strain, a total of 323,000 particles were extracted from 8,500 micrographs, 124k particles were selected to reconstruct map for the OMCC. For the OMCC from TraV/K/B-producing strain, a total of 321,000 particles were extracted from 5,500 micrographs, 65k particles were selected to reconstruct map for the OMCC from TraV/K/B-producing strain. Sample size were those required for the resolution.
Data exclusions	CryoEM micrographs with drifts and defocus >3 or <0.5 micrometer were excluded. extracted junk particles and broken/demaged/incomplete particles were excluded.
Replication	We did 3 independently purification experiments, and then we imaged the corresponding samples separately. We processed all the 3 datasets separately and successfully. All resulting maps are consistent. The data and results are replicable. During data processing, the dataset was randomly split into two halves, further ensuring reproducitibility.
Randomization	The data collection was randomly performed by EPU software. During data processing of image alignment, classification and reconstruction, the particle images were randomly split into two independent groups by cryoSparc software. The final resolution was accessed by FSC of the independent maps resulted from the two groups of datasets.
Blinding	The cryoEM data were blindly collected. The investigators were blind to data processing using cryoSparc software. The software was blind as no reference model was provided.

Reporting for specific materials, systems and methods

We require information from authors about some types of materials, experimental systems and methods used in many studies. Here, indicate whether each material, system or method listed is relevant to your study. If you are not sure if a list item applies to your research, read the appropriate section before selecting a response.

Materials & experimental systems

n/a	Involved in the study	
[ ]	[x]	Antibodies
[x]	[ ]	Eukaryotic cell lines
[x]	[ ]	Palaeontology and archaeology
[x]	[ ]	Animals and other organisms
[x]	[ ]	Human research participants
[x]	[ ]	Clinical data
[x]	[ ]	Dual use research of concern

Methods

n/a	Involved in the study	
[x]	[ ]	ChIP-seq
[x]	[ ]	Flow cytometry
[x]	[ ]	MRI-based neuroimaging

Antibodies

Antibodies used	Mouse monoclonal antibodies against the beta subunit of E. coli RNA polymerase were from BioLegend Clone: 8$B13, Isotype IgG2b, k; Cat: 663903. Lot: B287124. Polyclonal antibodies against the TraA pilin protein encoded by pED208. Raised by W. Paranchych and L. Frost, received as a gift from L. Frost. Described in publication listed in Validation section
Validation	Anti-RNP: Validation statement is on the Vendor's website. Reference cited in the validation report: Bergendahl V, et al. 2003. Protein Expr. Purif. 31:155.<br>2. Burgess RR and Thompson NE. 2002. Curr. Opin. Biotechnol. 13:304.<br>3. Stalder, ES. et al. 2011. Protein Expr. Purif. 77(1):26-33. (Epitope, ELISA, IP, WB)<br>The polyclonal anti-TraA pilin antibody was validated by L. Frost and W. Paranchych, as reported in Worobec et al. J. Bacteriol. 1986 167:660-665. IgG fraction was purified from rabbit serum, and reactivity to TraA pilin was confirmed by ELISA, immunoblot (Western) analysis, and immunogold labeling.
